# Beyond Adiposity: Lean Mass and Bone Mineral Content as Markers of Muscle Weakness and Physical Performance in Older Adults

**DOI:** 10.3390/medicina62040684

**Published:** 2026-04-03

**Authors:** Yeny Concha-Cisternas, Eduardo Guzmán-Muñoz, Walter Sepúlveda Loyola, Lincoyán Fernández Huerta, Felipe Montalva Valenzuela, Exal Garcia-Carrillo, Iván Molina Márquez, Rodrigo Yañez-Sepúlveda

**Affiliations:** 1 Escuela de Kinesiología, Facultad de Salud, Universidad Santo Tomás, Talca 3460000, Chile; 2 Vicerrectoría de Investigación e Innovación, Universidad Arturo Prat, Iquique 1100000, Chile; 3 Escuela de Pedagogía en Educación Física, Facultad de Educación, Universidad Autónoma de Chile, Talca 3460000, Chile; 4 Faculty of Health and Social Sciences, Center for Research in Biological and Chemical Sciences, Universidad de Las Américas (UDLA), Santiago 7500975, Chile; wsepulveda@udla.cl; 5 Escuela de Kinesiología, Facultad de Ciencias de la Rehabilitación y Calidad de Vida, Universidad San Sebastián, Concepción 4030000, Chile; lincoyan.fernandez@uss.cl; 6 Escuela de Entrenador en Actividad Física y Deporte, Facultad de Ciencias Humanas, Universidad Bernardo O’Higgins, Santiago 8370040, Chile; felipemontalva95@gmail.com; 7 Department of Physical Activity Sciences, Faculty of Education Sciences, Universidad Católica del Maule, Talca 3480112, Chile; exal.garcia@gmail.com; 8 Department of Physical Activity Sciences, Universidad de Los Lagos, Osorno 5290000, Chile; 9 Facultad de Educación, Pedagogía en Educación Física, Universidad Adventista de Chile, Chillán 3780000, Chile; ivanmolina@unach.cl; 10 Programa de Doctorado en Ciencias de la Actividad Física Universidad, Católica del Maule, Talca 34809112, Chile; 11 Facultad de Educación y Ciencias Sociales, Universidad Andrés Bello, Viña del Mar 2200055, Chile; rodrigo.yanez.s@unab.cl; 12 School of Medicine, Universidad Espíritu Santo, Samborondón 092301, Ecuador

**Keywords:** body composition, bone mineral content, adiposity, physical performance, muscle weakness

## Abstract

*Background and Objectives:* The contribution of body composition to muscle weakness and physical performance in older adults remains incompletely defined. This study aimed to evaluate the discriminative capacity of total and segmental body composition variables to identify muscle weakness and low physical performance in older adults. *Materials and Methods:* A cross-sectional study was conducted in 268 community-dwelling older adults (72.2 ± 8.2 years; 81.3% women). Body composition (lean mass, fat mass, and bone mineral content [BMC], total and segmental) was assessed using dual-energy X-ray absorptiometry. Muscle weakness was assessed by handgrip strength (≤27 kg in men; ≤16 kg in women), and low physical performance by the Short Physical Performance Battery ≤8. Sex-stratified receiver operating characteristic (ROC) analyses were performed. *Results:* No significant differences were found between sexes for age (*p* = 0.307) or body mass index (*p* = 0.892). However, men exhibited significantly higher waist circumference (105.2 ± 11.9 vs. 97.8 ± 12.4 cm; *p* < 0.001) and handgrip strength (30.3 ± 6.8 vs. 18.3 ± 4.6 kg; *p* < 0.001) than women. Regarding body composition, men presented higher total lean mass (50.4 ± 6.9 vs. 37.2 ± 4.6 kg; *p* < 0.001) and total bone mineral content (2666 ± 483 vs. 1940 ± 286 g; *p* < 0.001). *Conclusions:* Body composition variables showed higher discriminative capacity for muscle weakness than for low physical performance. The ability of lean mass and BMC to identify low physical performance was modest in both sexes, suggesting that structural body composition variables alone may be insufficient to discriminate complex functional impairment in older adults.

## 1. Introduction

Biological aging is characterized by progressive alterations in the architecture and composition of body tissues, including reductions in skeletal muscle mass, loss of bone mineral content (BMC), and significant redistribution of body adiposity [1,2,3]. These structural alterations compromise the integrity of the musculoskeletal system by impairing both the contractile capacity of skeletal muscle and the mechanical support provided by the skeleton, thereby affecting force generation and transmission, postural stability, and movement efficiency [4,5]. Collectively, these changes manifest clinically as sarcopenia, a syndrome defined by reduced muscle strength, decreased lean mass, and impaired physical performance, which is associated with an increased risk of adverse outcomes such as frailty, disability, hospitalization, and mortality [6,7,8]. Concurrently, increased adiposity, frequently accompanied by intramuscular fat infiltration, may coexist with a decline in lean mass, giving rise to phenotypes of sarcopenic obesity that further exacerbate functional deterioration [9,10,11,12].

Given the substantial burden of morbidity and mortality associated with these phenotypes, the early and timely detection of functional impairment is essential. Accordingly, the assessment of muscle strength and physical performance has become a central component of geriatric evaluation and epidemiological research [13,14]. On the one hand, handgrip strength is widely recognized as a simple, reproducible, and valid marker of systemic muscle weakness. It constitutes the primary criterion for identifying probable sarcopenia according to the European Working Group on Sarcopenia in Older People (EWGSOP2) consensus [14,15]. On the other hand, instruments such as the Short Physical Performance Battery (SPPB) enable objective assessment of physical performance through standardized tasks that evaluate balance, chair-rise performance, and gait speed. This tool has demonstrated robust predictive validity for disability, institutionalization, and mortality in older populations [16,17].

However, these assessments robustly quantify the functional phenotype but do not enable identification of the specific contributions of individual body compartments to the observed decline. Evidence suggests that body composition plays a relevant role in this process. A study of men aged 70 years or older reported that those with low physical performance exhibited lower segmental lean mass in both the upper and lower extremities, higher total adiposity, and significantly reduced muscle strength, as reflected by lower handgrip and quadriceps strength [18]. Similarly, another study demonstrated that older adults with higher physical performance and greater handgrip strength displayed more favorable body composition profiles, characterized by lower central adiposity and higher appendicular lean mass [19]. In contrast, Orwoll et al. [20] observed that only lower muscle mass was independently associated with poorer physical performance, whereas total body adiposity showed weak or non-significant associations.

Regarding the skeletal component, previous studies indicate that impaired mineral status, commonly assessed by bone mineral density, is associated with lower muscle strength and poorer physical performance in older adults [21]. These findings are consistent with the concept of the musculoskeletal unit and the functional biomechanical coupling between muscle and bone. However, research has focused predominantly on bone mineral density, leaving the role of BMC comparatively underexplored [22]. Unlike BMD, which reflects mineral concentration per unit area, BMC represents the absolute mineral mass of the skeleton and may more accurately capture the overall structural reserve of bone tissue, thereby constituting a potential functional marker that remains to be fully characterized [22]. Although the literature links individual components of body composition with physical performance and muscle weakness, the available evidence remains heterogeneous. Many studies rely on global indices that simultaneously integrate muscle, fat, and bone, thereby limiting the ability to disentangle the specific contribution of each compartment [23,24]. Consequently, it remains unclear whether functional decline is predominantly attributable to increased adiposity, reduced lean mass, or alterations in the skeletal component, and whether the regional distribution of these tissues differentially affects functional capacity [24,25]. This uncertainty is further compounded by the inherent sexual dimorphism in body composition, which restricts the clinical extrapolation of findings when rigorous sex-stratified analyses are not performed.

While previous studies have established general associations between body mass components and physical function, there is a notable scarcity of research utilizing receiver operating characteristic (ROC) curve analyses to derive specific cut-off points for older adults, particularly when combining segmental DXA-derived measures with sex-stratified analytical approaches. Establishing precise thresholds—especially for underexplored variables such as regional lean mass and bone mineral content—is essential to enhance the clinical utility of DXA in screening for functional impairment. Therefore, this study aimed to evaluate the discriminative capacity of body composition variables, including total and regional lean mass, fat mass, and BMC, in identifying muscle weakness and low physical performance in older adults, using sex-stratified analyses. Based on the concept of the musculoskeletal unit and prior evidence linking structural body composition to functional capacity in ageing, we hypothesized that lean mass and bone mineral content would have greater discriminative capacity for identifying muscle weakness and low physical performance than adiposity-related variables.

## 2. Materials and Methods

### 2.1. Study Design and Participants

This observational, cross-sectional study employed a non-probability convenience sampling method. The final sample comprised 268 community-dwelling older adults (50 men and 218 women) residing in the city of Talca, Chile, who were actively enrolled in local senior centers. Recruitment was conducted through these centers, and the resulting sex distribution reflects the typical demographic composition of community senior programs, in which female participation is substantially higher than male participation.

Inclusion Criteria: Participants were eligible if they met the following criteria: (a) age between 60 and 90 years; (b) optimal cognitive level to understand instructions, evaluated with the Mini-Mental State Examination (MMSE ≥ 14 points) [26]; and (c) functional independence, classified as “independent” or “independent with risk” according to the Functional Examination of the Elderly (EFAM-Chile) [27].

Exclusion Criteria: Participants were excluded if they: (a) used assistive mobility devices (walkers, slings, crutches, canes, or other support devices); (b) presented physical limitations, acute injuries, or severe sensory impairments (blindness or deafness) that prevented the performance of physical tests; or (c) had metallic implants (e.g., hip replacements) that could interfere with the body composition analysis.

Before data collection, all participants were thoroughly informed about the study’s background, methodology, and objectives to ensure complete understanding, and they provided written informed consent. Participation was entirely voluntary. The research was approved by the local Ethics Committee and adhered to the ethical guidelines outlined in the Declaration of Helsinki regarding studies involving human subjects.

### 2.2. Outcomes

#### 2.2.1. Anthropometric and Body Composition Assessment

Body mass index (BMI) was calculated as body weight (kg) divided by height squared (m^2^). Body weight was obtained with a calibrated electronic scale (Seca, Hamburg, Germany) with participants wearing light clothing and no shoes. The scale had a precision of 0.1 kg. Additionally, waist circumference (cm) was measured to the nearest 0.1 cm using a flexible, non-elastic tape (Seca 201, Reinach, Switzerland) at the midpoint between the lowest rib and the iliac crest [28].

Subsequently, body composition was evaluated using dual-energy X-ray absorptiometry (DXA) with a GE Lunar Prodigy scanner (General Electric Healthcare, Madison, WI, USA) and Encore software version 13.60. The scanner was calibrated daily using a standard phantom block to ensure measurement stability (coefficient of variation < 1.0%). Participants were scanned in the supine position, wearing light clothing and without metal objects, in accordance with International Society for Clinical Densitometry (ISCD) guidelines [29]. The software automatically segmented the body into anatomical regions (arms, legs, trunk, and total body). For this study, the following variables were derived for each segment and reported as continuous outcomes: Lean Soft Tissue Mass (kg), calculated as fat-free mass minus bone mineral content; Bone Mineral Content (BMC, g); absolute Fat Mass (kg); and Body Fat Percentage (%), representing the proportion of fat mass relative to total tissue mass.

#### 2.2.2. Muscle Strength

Muscle strength was assessed using a handgrip strength test with a calibrated digital dynamometer (Takei TKK5401, Niigata, Japan). Participants performed the test in a seated position of triple flexion (hips, knees, and ankles at 90°), with the shoulder adducted, the elbow flexed at 90°, and the forearm in a neutral position [30]. Three maximal isometric contractions were performed, with each effort maintained for 3 s using the dominant hand [31]. A one-minute resting interval was provided between attempts to prevent muscle fatigue. The maximum value (kg) obtained from the three trials was used for analysis. Values ≤ 27 kg for men and ≤16 kg for women were considered as muscle weakness, based on the EWGSOP2 criteria [15].

#### 2.2.3. Physical Performance

Physical Performance was measured using the Short Physical Performance Battery (SPPB). This battery comprises three timed sub-tests, each scored from 0 (worst) to 4 (best): standing balance (ability to maintain side-by-side, semi-tandem, and full-tandem stances for 10 s), gait speed (time to walk 4 m at usual pace, best of two trials), and the chair stand test (time to complete five consecutive chair rises). The total score ranges from 0 to 12. A total score of ≤8 points was operationally defined as Low Physical Performance to identify participants with functional limitations [15].

### 2.3. Statistical Analysis

Data analysis was conducted using the Python (v3.13) statistical environment (Pandas library v1.3 and SciPy v1.7). In the initial stage, an exploratory analysis was performed to identify outliers and verify the internal consistency of the variables. The distribution of continuous variables was assessed through visual inspection of histograms and examination of skewness and kurtosis. Additionally, the Kolmogorov–Smirnov test was formally used to assess normality. The results indicated that the variables did not deviate significantly from normality (*p* > 0.05). Given the sample size (n = 268), independent-samples *t*-tests were considered appropriate because parametric tests are robust to moderate departures from normality. Continuous variables are presented as mean ± standard deviation (SD), along with their respective 95% confidence intervals (95% CI). Differences in anthropometric, body composition, and functional characteristics between men and women were evaluated using independent-samples t-tests.

To determine the discriminative capacity of body composition variables (lean mass, fat mass, and bone mineral content across body segments) for identifying Muscle Weakness and Low Physical Performance, Receiver Operating Characteristic (ROC) curve analyses were conducted. ROC curve analyses were performed using unadjusted body composition variables to evaluate their discriminative capacity for each outcome. The Area Under the Curve (AUC) was calculated along with its corresponding 95% confidence intervals (95% CI). Optimal cut-off points for each variable were identified using the Youden Index (J = sensitivity + specificity − 1), selecting the threshold that jointly maximized sensitivity and specificity. A *p*-value < 0.05 was considered statistically significant for between-sex comparisons.

## 3. Results

Table 1 summarizes the participants’ demographic, anthropometric, functional, and body composition characteristics. No significant differences were observed in age (73.1 ± 6.9 vs. 72.0 ± 8.5 years; *p* = 0.307) or body mass index (29.7 ± 4.8 vs. 29.8 ± 5.0 kg/m^2^; *p* = 0.892). However, men were significantly heavier (81.5 ± 15.6 vs. 69.1 ± 12.3 kg; *p* < 0.001) and presented a larger waist circumference (105.2 ± 11.9 vs. 97.8 ± 12.4 cm; *p* < 0.001). Regarding functional assessments, men exhibited significantly higher handgrip strength (30.3 ± 6.8 vs. 18.3 ± 4.6 kg; *p* < 0.001), while no significant differences were found in the SPPB total score (10.0 ± 2.5 vs. 9.8 ± 2.1 points; *p* = 0.698) or gait speed (3.4 ± 1.1 vs. 3.3 ± 0.9 points; *p* = 0.286). Consistent sex-specific differences were confirmed in body composition: men displayed greater total lean mass (50.4 ± 6.9 vs. 37.2 ± 4.6 kg; *p* < 0.001) and total bone mineral content (2666 ± 483 vs. 1940 ± 286 g; *p* < 0.001). While women presented a higher total body fat percentage (43.7 ± 5.8% vs. 35.1 ± 7.5%; *p* < 0.001), absolute total fat mass did not differ significantly between sexes (27.7 ± 9.6 vs. 29.8 ± 8.7 kg; *p* = 0.158).

Regarding functional outcomes, among men (n = 50), 16 participants (32.0%) met the criteria for muscle weakness, and 10 (20.0%) had low physical performance (SPPB ≤ 8). Among women (n = 218), 68 participants (31.2%) had muscle weakness, and 51 (23.4%) had low physical performance.

Table 2 presents the discriminative capacity of body composition variables for identifying muscle weakness and low physical performance (SPPB ≤ 8), stratified by sex. Regarding muscle weakness in men, multiple indicators exhibited good discriminative capacity (AUC = 0.77–0.81). Notably, Trunk Lean Mass (<22.4 kg) demonstrated specificity of 91% and sensitivity of 63%. Total Lean Mass (<45.5 kg) and Leg BMC (<900 g) showed similar performance, with high specificity (88%) and sensitivities ranging from 62% to 69%. In women, Total BMC, Arm Lean Mass, and Arm BMC showed AUCs of 0.66, with sensitivities ranging from 58% to 74% and specificities ranging from 59% to 69%. Notably, adiposity measures (absolute and percentage) showed overall limited discriminative capacity across outcomes.

**Figure 1:**
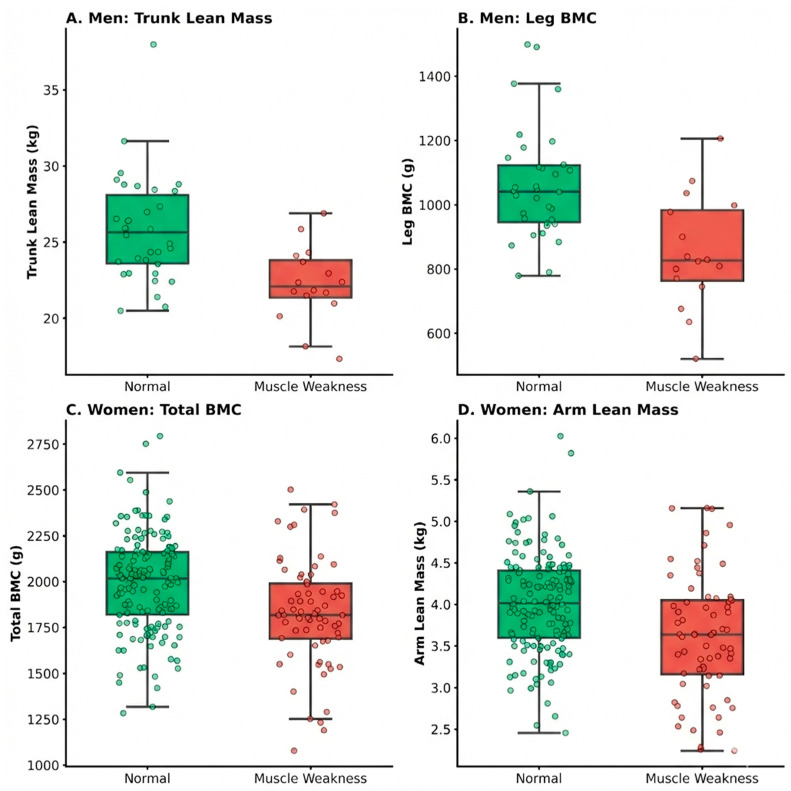
Comparison of key body composition variables.

Figure 1 illustrates the distribution of key body composition variables stratified by muscle weakness status. In men, participants classified as having muscle weakness exhibited significantly lower Trunk Lean Mass and Leg BMC than the normal strength group (Figure 1A,B), with a clear separation between the interquartile ranges. In women, a similar trend was observed for Total BMC and Arm Lean Mass (Figure 1C,D), although the overlap between groups was more pronounced than in men.

**Figure 2:**
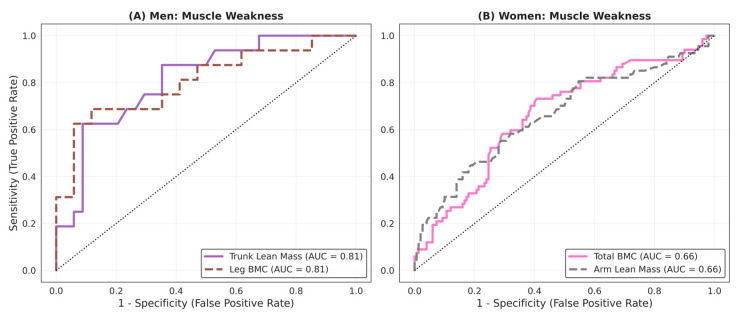
ROC curves for identifying muscle weakness.

The receiver operating characteristic (ROC) curve analysis (Figure 2) showed that body composition parameters exhibited higher discriminative capacity for muscle weakness in men than in women, where the discriminative capacity was moderate. In men, trunk lean mass and leg bone mineral content (BMC) showed excellent discriminative performance, with AUCs of 0.81. The optimal cutoff values were <22.4 kg for trunk lean mass (sensitivity 63%, specificity 91%) and <900 g for leg BMC (sensitivity 69%, specificity 88%). Total lean mass also showed high discriminative capacity (AUC = 0.81; cut-off <45.5 kg).

In women, the associations were weaker. The variables with the highest discriminative capacity were total BMC and arm lean mass, both with an AUC of 0.66. The optimal threshold for total BMC was <1947 g (sensitivity 74%, specificity 59%). Although some adiposity measures reached AUC values slightly above 0.60, their overall discriminative capacity was modest.

**Figure 3:**
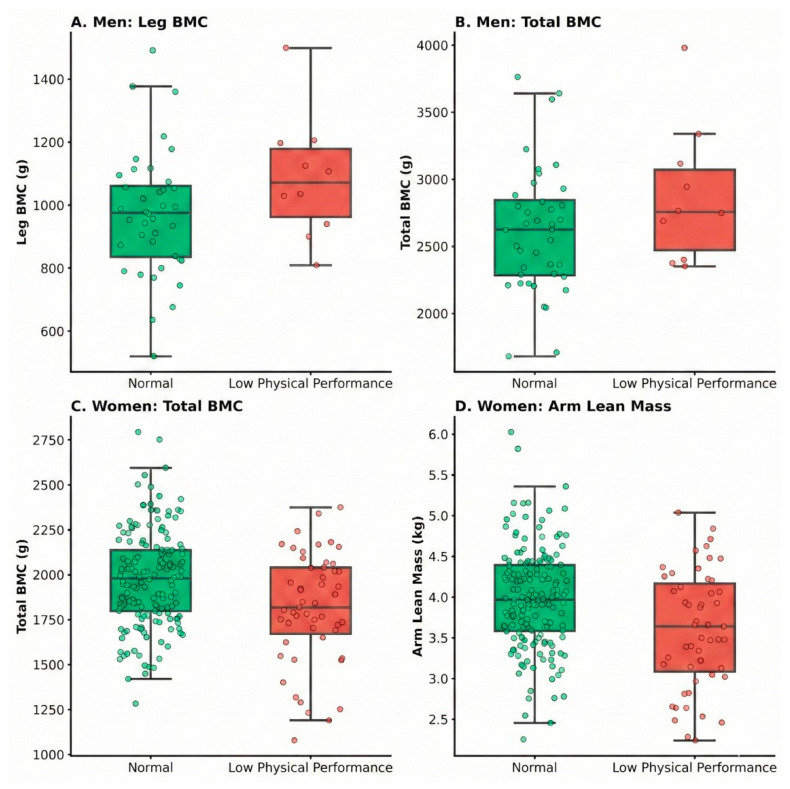
Comparison of body composition variables by physical performance.

**Figure 4:**
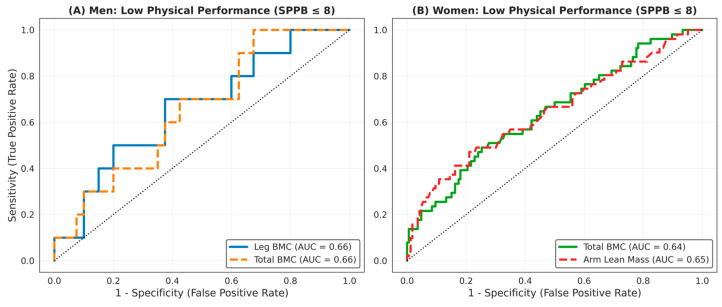
ROC curves for identifying low physical performance.

Regarding physical performance, Figure 3 illustrates the body composition profiles of participants with low physical performance (SPPB ≤ 8). Men with low physical performance exhibited reduced leg and total BMC (Figure 3A,B). Similarly, women with low physical performance had lower total BMC and arm lean mass (Figure 3C,D). However, as demonstrated by the ROC analysis (Figure 4), the discriminative capacity of body composition variables for identifying low physical performance was lower than that observed for muscle weakness. In men, leg BMC and total BMC each yielded an AUC of 0.66 (Figure 4A), whereas in women, total BMC and arm lean mass reached AUCs of 0.64 (Figure 4B). These findings suggest that although reductions in lean and bone mass are associated with functional impairment, their independent discriminative ability for complex physical performance tasks is limited compared to their stronger association with muscle weakness.

## 4. Discussion

The principal finding of this study was that the discriminative capacity of body composition varies substantially according to the functional outcome assessed and sex. While lean mass and BMC demonstrated the highest discriminative capacity for muscle weakness, particularly in men, their ability to identify low physical performance was modest in both sexes. Additionally, a relevant finding was the lack of discriminative utility of total or segmental adiposity in identifying low muscle strength and low physical performance. These results challenge the traditional view of fat mass as a primary determinant of functional decline in older age.

Specifically, when analyzing muscle weakness, lean mass and BMC variables demonstrated the highest discriminative capacity, particularly in men, for whom trunk lean mass, total lean mass, and leg BMC achieved AUC values approaching 0.80. This finding is supported by Tavares et al. [22] in the ELSA-Brazil cohort, who demonstrated that both muscle mass and BMC act as independent and additive predictors of handgrip strength, reflecting the integrity of the muscle–bone unit. Similarly, Hauger et al. reported significant associations between lower-limb bone mass and handgrip strength in older adults of both sexes [32].

The discriminative capacity of lean mass and bone mineral content observed in our study is consistent with recent research using ROC analyses to define cut-off points for muscle weakness [33]. Previous investigations have demonstrated that DXA-derived lean mass indices can yield high discriminative capacity—with AUC values frequently exceeding 0.80—for identifying structural and functional decline in older populations [34]. Our results extend this evidence by showing that regional parameters, specifically trunk lean mass (<22.4 kg) and leg BMC (<900 g) in men, offer highly accurate cut-offs for discriminating low handgrip strength. However, these thresholds should be interpreted with caution, particularly in men, given the limited number of events, and should be considered exploratory until validated in larger and more balanced samples. In ROC analyses, a small number of events can increase the variability of AUC estimates and influence the stability of the derived cut-off points. Furthermore, our ROC models revealed limited discriminative capacity for adiposity, consistent with other studies that have reported difficulty identifying robust fat mass thresholds for discriminating functional performance, thereby suggesting that structural tissue quantity (muscle and bone) may represent a more informative clinical indicator [35]. However, the literature has also reported heterogeneous findings, particularly among women, in whom associations between body composition and muscle strength tend to be weaker or inconsistent [36,37].

It is noteworthy that although muscle weakness was assessed using handgrip strength, the most consistent associations were observed with lower-limb bone mass rather than with arm BMC, as might have been initially expected. This pattern suggests that handgrip strength is more strongly associated with total lean mass and the overall status of the musculoskeletal system than with a single anatomical compartment [22,24]. In this regard, the evidence supports interpreting handgrip strength as a global functional marker rather than as an exclusively localized measure of upper-limb function [22,24].

In the regional analysis, the ability of trunk lean mass and leg BMC to discriminate muscle weakness in the present study should therefore be interpreted in light of their role as indicators of overall musculoskeletal health rather than as direct mechanical determinants of handgrip strength. The trunk contains a substantial proportion of total lean mass. It plays a critical role in proximal stabilization, which has been associated with higher levels of global muscle strength in older adults [38,39]. Similarly, adequate leg BMC reflects the structural integrity of the musculoskeletal system, which in turn has been linked to greater force production capacity [40]. Therefore, the observed association between these regional variables and muscle weakness, as assessed by handgrip strength, does not imply a direct anatomical relationship; rather, it indicates that individuals with better overall muscle–bone status tend to exhibit greater strength, even in localized functional tests such as the one used in this study.

In women, the discriminative capacity of body composition was moderate, and the observed associations were weaker than those identified in men. This finding is consistent with previous research reporting a lower correspondence between body mass components and muscle strength in older women, which has been suggested to relate to a narrower absolute range of lean mass, sex-specific differences in the regional distribution of muscle and bone tissue, and a greater influence of non-structural factors [22]. In this context, our findings may support the notion that, in women, muscle weakness may depend on a broader set of physiological and clinical determinants, including neuromuscular activation, hormonal changes, motor coordination, comorbidities, and metabolic status. These factors may attenuate the independent contribution of body composition variables, thereby limiting their ability to discriminate muscle weakness when considered in isolation [3,41,42,43].

Another relevant finding of this study was that, in both sexes, body composition variables demonstrated only modest discriminative capacity for low physical performance (AUC ≈ 0.64–0.66). Despite these modest values, distinct segmental patterns emerged that provide relevant insight. In men, the body composition variables with the highest discriminative ability for low physical performance corresponded exclusively to the skeletal component, particularly total and leg BMC. In women, total and trunk BMC, together with upper-limb lean mass, showed the highest discriminative capacity, with AUC values around 0.64. However, some ROC-derived thresholds presented a sensitivity–specificity trade-off that may limit their clinical applicability. For instance, although the total BMC cut-off for identifying low physical performance in men showed very high sensitivity, its relatively low specificity indicates that such thresholds should be interpreted cautiously for clinical screening. These findings are consistent with previous studies reporting associations between body composition and physical performance assessed using the SPPB [3,19]. Indeed, structural variables such as BMC and appendicular lean mass have been identified as determinants of mobility and balance performance. However, their effects are typically moderate and may vary according to sex [21,44].

Interestingly, this study also identified a limited discriminative capacity of absolute and relative adiposity, both total and segmental, for identifying muscle weakness and low physical performance. Numerous studies have shown that higher adiposity (particularly central or lower-limb adiposity) is associated with poorer physical performance in older adults [19,45]. This association does not necessarily translate into adequate discriminative ability for identifying individuals with functional impairment. In this regard, quantitative measures of fat mass, whether global (e.g., body mass index or waist circumference) or segmental (e.g., total fat mass, percentage body fat, and trunk or limb fat mass), appear to have limited utility as isolated variables of poor functional performance [46]. The deleterious effects of adiposity on strength and physical performance have been proposed to operate primarily through qualitative alterations in muscle tissue, including intramuscular fat infiltration, impaired contractile quality, and chronic low-grade systemic inflammation—processes not captured by total or segmental fat mass measurements [47,48]. Consequently, adiposity may indirectly influence functional capacity through its interaction with skeletal muscle, which may explain its lower discriminative capacity relative to lean mass and BMC [49,50]. In this context, assessing muscle quality and the interaction between adipose and lean tissue, rather than relying solely on quantitative measures of body fat, may provide a more appropriate framework for understanding the role of adiposity in functional decline among older adults [51].

### Strengths and Limitations

This study has several strengths. Body composition was assessed using DXA, widely recognized as a reference method (gold standard) for quantifying lean mass, fat mass, and BMC, with high precision and reproducibility in estimating distinct tissue compartments. In addition, segmental analysis of the trunk and extremities enabled identification of the specific contributions of localized tissues, overcoming the limitations of global indicators such as body mass index or total adiposity, which do not distinguish among anatomical compartments. This approach was complemented by assessing clinically relevant functional outcomes, including handgrip strength and the SPPB, thereby enabling evaluation of both force-generating capacity and overall physical performance. Finally, sex-stratified analyses and receiver operating characteristic (ROC) curve modeling facilitated a clinically interpretable assessment of the discriminative capacity of each variable. They enabled the proposal of cutoff values with potential utility for the early detection of muscle weakness and low physical performance.

However, several limitations should be considered when interpreting these findings. The cross-sectional design precludes establishing causal or temporal relationships between body composition and physical function. Additionally, the use of a non-probabilistic convenience sampling strategy may introduce potential selection bias, as participants were recruited from community senior centers and may represent a relatively active and socially engaged subgroup of the older population. Consequently, the generalizability of the findings to the broader population of community-dwelling older adults should be interpreted with caution. The study sample also showed a marked sex imbalance, with a predominance of women, which reflects the typical demographic composition of community senior centers from which participants were recruited. Nevertheless, this imbalance may influence the stability and precision of sex-stratified analyses. In particular, the relatively small sample size of men (n = 50) resulted in a limited number of cases presenting muscle weakness (n = 16) and low physical performance (n = 10), which may affect the robustness of ROC-derived estimates. In discriminative capacity analyses, a small number of events can lead to wider confidence intervals and instability in identifying optimal cutoff points. Therefore, although the AUC values observed in men suggest a meaningful association between structural body composition parameters and functional outcomes, these thresholds should be interpreted cautiously and considered exploratory rather than clinically applicable until confirmed in larger, more balanced samples. Additionally, no internal or external validation procedures were performed for the derived cut-off values; therefore, these thresholds should be considered preliminary and require confirmation in independent populations. Furthermore, although DXA accurately quantifies tissue mass, it does not assess qualitative features such as intramuscular fat infiltration, muscle quality, or neuromuscular activation, which may contribute to functional variability not explained solely by body mass. Finally, the analytical approach focused on evaluating the discriminative capacity of body composition variables using ROC analysis rather than on estimating independent causal associations; therefore, potential confounding factors were not explicitly accounted for in the present study.

## 5. Conclusions

This study demonstrates that, even when employing detailed segmental body composition analysis with DXA and accounting for sex differences, the discriminative capacity of structural body composition variables varies with the functional outcome assessed. Lean mass and BMC showed greater ability to discriminate muscle weakness, whereas their performance for detecting low physical performance was modest in both sexes. Furthermore, adiposity measures, whether total or segmental, showed limited discriminative capacity for both outcomes. However, these findings should not be interpreted as evidence of a lack of pathophysiological relevance of adiposity, as its effects on functional outcomes may involve mechanisms not captured by the present analytical approach. Overall, these results suggest that structural body composition variables alone may be insufficient to distinguish complex physical performance impairments in older adults.

## Figures and Tables

**Table 1:** Descriptive, functional, and body composition characteristics of the study participants stratified by sex.

Outcome	Total (n = 268)	Men (n = 50)	Women (n = 218)	*p*-Value
Age (years)	72.2 ± 8.2 [71.2–73.2]	73.1 ± 6.9 [71.2–75.0]	72.0 ± 8.5 [70.9–73.1]	0.307
Weight (kg)	71.4 ± 13.8 [69.8–73.1]	81.5 ± 15.6 [77.1–85.8]	69.1 ± 12.3 [67.5–70.7]	<0.001
Height (m)	1.55 ± 0.09 [1.54–1.56]	1.66 ± 0.09 [1.63–1.68]	1.52 ± 0.06 [1.51–1.53]	<0.001
BMI (kg/m^2^)	29.8 ± 5.0 [29.2–30.4]	29.7 ± 4.8 [28.4–31.0]	29.8 ± 5.0 [29.1–30.5]	0.892
Waist circumference (cm)	99.2 ± 12.7 [97.7–100.7]	105.2 ± 11.9 [102.0–109.0]	97.8 ± 12.4 [96.1–99.5]	<0.001
Handgrip (kg)	20.6 ± 6.9 [19.7–21.4]	30.3 ± 6.8 [28.5–32.2]	18.3 ± 4.6 [17.7–18.9]	<0.001
SPPB (points)	9.8 ± 2.1 [9.6–10.1]	10.0 ± 2.5 [9.3–10.7]	9.8 ± 2.1 [9.5–10.1]	0.698
Total Fat %	42.1 ± 7.0 [41.2–42.9]	35.1 ± 7.5 [33.0–37.2]	43.7 ± 5.8 [42.9–44.4]	<0.001
Trunk Fat %	45.5 ± 7.9 [44.6–46.5]	39.6 ± 8.5 [37.2–41.9]	46.9 ± 7.1 [45.9–47.8]	<0.001
Leg Fat %	39.1 ± 7.9 [38.1–40.0]	29.0 ± 6.8 [27.1–30.8]	41.4 ± 6.1 [40.6–42.2]	<0.001
Arm Fat %	41.2 ± 8.1 [40.2–42.2]	30.1 ± 7.3 [28.1–32.1]	43.7 ± 5.8 [43.0–44.5]	<0.001
Total Fat Mass (kg)	29.4 ± 8.9 [28.3–30.5]	27.7 ± 9.6 [25.0–30.3]	29.8 ± 8.7 [28.6–30.9]	0.158
Trunk Fat Mass (kg)	16.9 ± 5.6 [16.2–17.5]	17.1 ± 6.3 [15.3–18.8]	16.8 ± 5.4 [16.1–17.6]	0.822
Leg Fat Mass (kg)	8.5 ± 3.1 [8.1–8.9]	7.0 ± 2.9 [6.2–7.7]	8.9 ± 3.0 [8.5–9.3]	<0.001
Arm Fat Mass (kg)	3.0 ± 0.9 [2.9–3.1]	2.6 ± 1.0 [2.4–2.9]	3.1 ± 0.9 [3.0–3.2]	0.004
Total Lean Mass (kg)	39.7 ± 7.3 [38.8–40.5]	50.4 ± 6.9 [48.5–52.3]	37.2 ± 4.6 [36.6–37.8]	<0.001
Trunk Lean Mass (kg)	19.5 ± 3.7 [19.1–20.0]	24.7 ± 3.6 [23.7–25.7]	18.3 ± 2.5 [18.0–18.7]	<0.001
Leg Lean Mass (kg)	12.9 ± 2.6 [12.6–13.2]	16.5 ± 2.8 [15.7–17.2]	12.1 ± 1.8 [11.9–12.4]	<0.001
Arm Lean Mass (kg)	4.3 ± 1.1 [4.1–4.4]	6.0 ± 1.1 [5.7–6.3]	3.9 ± 0.7 [3.8–4.0]	<0.001
Total BMC (g)	2075 ± 435 [2023–2127]	2666 ± 483 [2532–2800]	1940 ± 286 [1902–1978]	<0.001
Trunk BMC (g)	665 ± 166 [645–685]	866 ± 194 [812–920]	619 ± 118 [604–635]	<0.001
Leg BMC (g)	708 ± 191 [685–731]	996 ± 199 [940–1051]	642 ± 112 [627–657]	<0.001
Arm BMC (g)	255 ± 69 [247–263]	362 ± 71 [342–382]	230 ± 38 [225–235]	<0.001

Data are presented as mean ± standard deviation [95% Confidence Interval]. BMI: Body Mass Index; WC: Waist Circumference; SPPB: Short Physical Performance Battery; BMC: Bone Mineral Content.

**Table 2:** Optimal cut-off points and discriminative capacity (AUC) of body composition variables for identifying Muscle Weakness and Low Physical Performance.

Condition	Sex	Variable	AUC (CI 95%)	Cut-Off	Sensitivity (%)	Specificity (%)
Muscle Weakness	Men	Trunk Lean Mass (kg)	0.81 [0.673–0.953]	<22.4	63	91
		Total Lean Mass (kg)	0.81 [0.673–0.953]	<45.5	62	88
		Leg BMC (g)	0.81 [0.663–0.947]	<900	69	88
		Arm BMC (g)	0.77 [0.619–0.921]	<314	69	91
		Leg Lean Mass (kg)	0.77 [0.619–0.921]	<15.3	69	82
	Women	Total BMC (g)	0.66 [0.583–0.745]	<1947	74	59
		Arm Lean Mass (kg)	0.66 [0.582–0.744]	<3.7	59	69
		Arm BMC (g)	0.66 [0.583–0.745]	<230	72	61
Low Physical Performance	Men	Leg BMC (g)	0.66 [0.462–0.864]	<1029	70	63
		Total BMC (g)	0.66 [0.453–0.857]	<2351	100	33
		Arm BMC (g)	0.61 [0.408–0.816]	<364	70	60
	Women	Arm Lean Mass (kg)	0.64 [0.555–0.735]	<3.5	49	77
		Trunk BMC (g)	0.64 [0.549–0.731]	<538	43	81
		Total BMC (g)	0.64 [0.550–0.732]	<1804	49	74

AUC: Area Under the Curve; BMC: Bone Mineral Content; SPPB: Short Physical Performance Battery. The cut-off symbol (<) denotes the threshold below which the participant is identified as having the condition (Muscle Weakness or Low Physical Performance). Sensitivity and Specificity are expressed as percentages. [95% Confidence Interval].

## Data Availability

The data that support the findings of this study are available from the corresponding author upon reasonable request.
